# Exploration of Shoulder Abscess Association With Prompt Aggregatibacter aphrophilus Growth in Infective Endocarditis

**DOI:** 10.7759/cureus.23107

**Published:** 2022-03-12

**Authors:** Sina Bagheri, Nene Takahashi, Victoria R Ramirez, Deepthi K Jayasekara

**Affiliations:** 1 College of Osteopathic Medicine of the Pacific, Western University of Health Sciences, Pomona, USA; 2 Infectious Disease, Emanate Health Queen of the Valley Hospital, West Covina, USA

**Keywords:** duke's criteria, trans-thoracic echocardiogram, transesophageal echocardiography (tee), intracranial hemorrhage, septic emboli, superficial abscess, infective endocarditis, hacek

## Abstract

*Aggregatibacter aphrophilus*, formerly known as *Haemophilus aphrophilus*, is one member of a group of bacteria referred to as HACEK (*Haemophilus*, *Aggregatibacter*, *Cardiobacterium*, *Eikenella*, *Kingella*) organisms. Infections from any of the HACEK organisms typically lead to very poor outcomes and can be difficult to manage, especially when complicated by intracranial hemorrhage (ICH). HACEK organisms can also be difficult to grow on blood cultures, and *A. aphrophilus* is rarely seen, if at all. Traditionally, most laboratories follow an extended incubation protocol of 14 to 21 days to aid the growth of HACEK bacteria. Herein we report a case of infective endocarditis where *A. aphrophilus* resulted on blood culture in three days, in a patient with a right shoulder abscess, complicated by septic embolization leading to ICH. We explore a potential link between the prompt growth of *A. aphrophilus* on blood culture and the presence of the right shoulder abscess.

## Introduction

*Aggregatibacter aphrophilus*, formerly known as *Haemophilus aphrophilus*, is a fastidious, Gram-negative coccobacillus that is one member of a group of bacteria referred to as HACEK (*Haemophilus*, *Aggregatibacter*, *Cardiobacterium*, *Eikenella*, *Kingella*) organisms [1]. *A. aphrophilus* is commonly associated with dental infections but has also been linked to cases of infective endocarditis (IE) [2]. Infections from any of the HACEK organisms typically lead to very poor outcomes and can be difficult to manage, especially when complicated by intracranial hemorrhage (ICH) [3]. These organisms typically colonize the oropharynx and tend to be very difficult to grow on blood cultures [4]. Due to the difficulty of isolation, this bacterium is rarely seen in blood cultures, if at all. Traditionally most laboratories follow an extended incubation protocol of 14 to 21 days in order to aid the growth of HACEK bacteria [5]. In this report, we explore a case where a blood culture grew *A. aphrophilus* much faster than expected. We wish to draw attention to a concurrent localized abscess with this presentation, as a possible explanation for the expedited results. 

Herein we report a case of IE caused by *A. aphrophilus* in a patient with a right shoulder abscess, complicated by septic embolization leading to ICH. In addition, we provide a review of the available literature on other reported cases of *Aggregatibacter*-induced acute IE.

## Case presentation

A 74-year-old man with a past medical history of hypertension and hyperlipidemia presented to the emergency department with complaints of nagging right shoulder pain, generalized weakness, chills, palpitations, and poor appetite. The patient stated that his right shoulder pain had been present for the past two months and this was the first time he sought out treatment for it. The patient stated that the right shoulder pain gradually increased over time, was rated 6/10, and did not radiate to any other part of his body. The patient denied current tobacco or alcohol use, but had a 20 pack-year history of smoking cigarettes and a history of heavy alcohol use. The patient stated that he quit smoking tobacco and drinking alcohol over five years ago

On initial presentation, the patient presented with a blood pressure of 107/49, heart rate of 140 beats/min, respiratory rate of 20 breaths/min, and temperature of 98.6°F. The patient’s temperature peaked at 99.4°F on the day of admission. The patient appeared moderately distressed, mainly due to his right shoulder pain. The physical examination was largely unremarkable with the exception of a 2 cm × 2 cm fluctuant mass on the right deltoid region. 

Electrocardiogram (ECG), culture of the mass, urine culture, blood cultures, and routine blood work were performed. The ECG showed the patient to be in atrial fibrillation with rapid ventricular response (RVR). Significant findings from the blood work revealed the white blood cell (WBC) count at 16,000/μL, bands at 15%, and lactate at 5.4 mmol/L. Empiric IV ampicillin/sulbactam 3 grams every 6 hours was initiated for the right shoulder abscess and concerns of sepsis. 

Based on the patient’s vital signs and blood test results, contrast-enhanced computed tomography (CT) of the abdomen was ordered to further investigate an additional source of infection causing sepsis. CT of the abdomen was negative for a source of infection. Additionally, a CT pulmonary angiogram (CTPA) with contrast was ordered given the patient’s weakness to rule out pulmonary embolism (PE). Findings from CTPA were negative for PE. Given the patient's new onset of atrial fibrillation with RVR, a transthoracic echocardiogram was performed and mild mitral and tricuspid regurgitation were noted. The patient denied having ever been seen by a cardiologist or having been informed regarding the presence of a murmur. 

On day 4, two separate blood and abscess cultures revealed *A.* *aphrophilus*. Synergistic gentamicin 1 mg/kg every 8 hours for five days was added to the antibiotic regimen for the bacteremia. The patient’s temperature was 96.8°F and WBC count decreased to 12,800/μL. Following the culture results, a definitive diagnosis of IE was made using Duke's criteria. The two major criteria that were fulfilled include typical microorganisms consistent with IE in two different blood cultures and novel regurgitation on echocardiogram. A transesophageal echocardiogram (TEE) was planned to further investigate IE.

**Figure 1:**
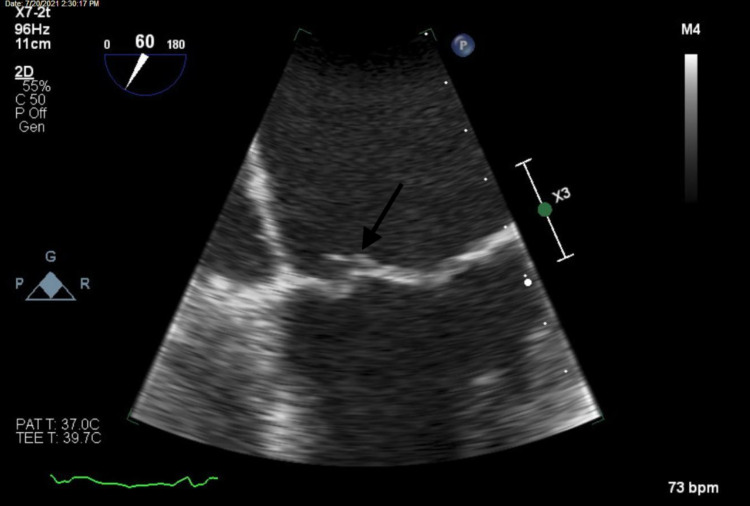
Transesophageal echocardiogram, midesophageal four-chamber view, revealing a small, mobile density attached to the anterior mitral valve leaflet.

On day 5, the patient was found to be awake and in no acute distress with a Glasgow Coma Scale (GCS) of 15. TEE was performed and showed a mildly thickened mitral valve leaflet with a mobile density attached, which raised concerns for a bacterial vegetation, as seen in Figure 1. Moderate mitral regurgitation was also noted that may have been underestimated due to the eccentric nature of the jet. The patient’s temperature was 98.7°F. 

**Figure 2:**
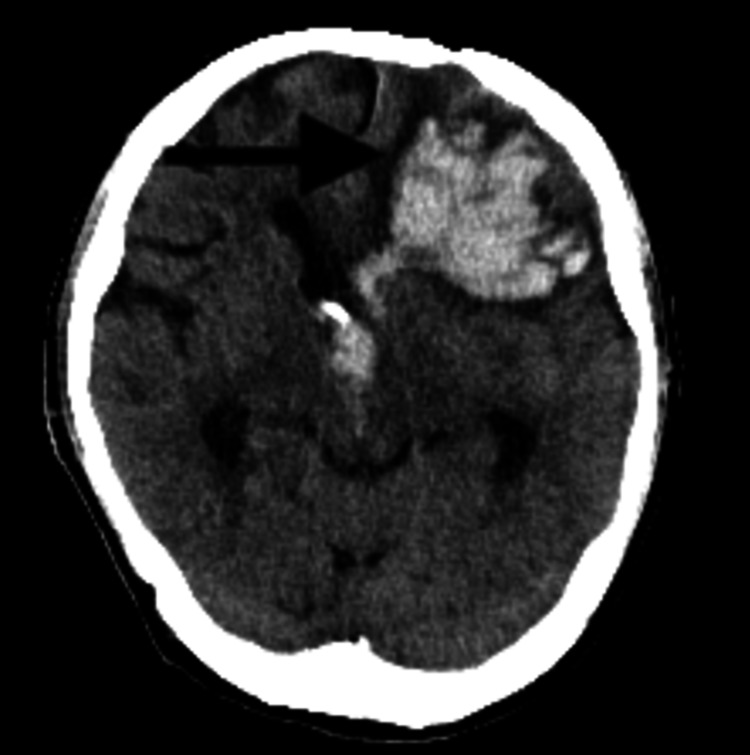
CT of the head/brain without contrast showing a large left frontal intraparenchymal hematoma measuring up to 5.7cm with surrounding edema and intraventricular hemorrhage within the lateral, third, and fourth ventricles. CT, computed tomography.

On day 6, the patient was found to be altered, unresponsive to painful stimuli, and with snoring respirations suspicious of a stroke with a GCS of 3. The patient was subsequently intubated and managed with a ventilator. CT of the head without contrast was performed and showed evidence of ICH as a likely result of hemorrhagic transformation following an embolic stroke, as seen in Figure 2. The results of the CT of the head also showed a large left intraparenchymal hematoma measuring 5.7 cm with surrounding edema with moderate hydrocephalus and hemorrhage within the lateral, third, and fourth ventricles. An external ventricular drain was placed to help facilitate a decrease in ventricular dilation. The patient remained in a critical state and required continued mechanical ventilation. Neurologically, the patient was unarousable with a GCS of 3T.

On day 8, the patient was mildly arousable off sedation, was able to follow simple commands, and responded to painful stimuli on the left side more than right with a GCS of 6T. The patient’s neurological status remained stable for the next week. Given the minimal clinical improvement, the decision was made to stabilize the patient with the placement of a tracheostomy tube and percutaneous endoscopic gastrostomy tube. Two sets of repeat blood cultures were performed and both resulted in no growth. The patient’s temperature was 96.8°F.

On day 15, the patient was able to open his eyes and speak in small sentences. Spontaneous breathing trials were performed to assess the patient's respiratory status. However, the patient’s mental status remained too poor to extubate. The patient’s neurological status improved significantly with a GCS of 11. After three weeks in the hospital, the patient was transferred to a long-term acute care hospital for the administration of ceftriaxone 2 grams daily for three more weeks, given the pan-antibiotic sensitivity of *A. aphrophilus*, and remains in stable condition. 

## Discussion

Acute IE is a disease with low prevalence but poor prognosis, and is most commonly caused by Gram-positive bacteria such as *Staphylococcus aureus, Streptococcus viridans*, or *Streptococcus bovis* [6]. The HACEK group of organisms is a rare etiological agent for IE, with only 1.4% to 3% of IE cases reported being due to these organisms [1]. Among the HACEK organisms, the *Aggregatibacter* genus is the most dominant etiology and is strongly associated with periodontitis [7]. In our literature review, we found one case where *A. aphrophilus* endocarditis had further spread to involve bone and soft tissue presenting as an abscess and osteomyelitis [8]. This finding further supports our hypothesis that the right shoulder abscess may be related to the patient’s endocarditis. 

Due to the difficulty of isolation of *A. aphrophilus* with conventional culture techniques, little data are present on the time it takes for a positive blood culture result. The latest available data addressing time to detection was a retrospective multicenter evaluation by Petti et al. In this study, an overall mean time to detection of an *Aggregatibacter* species was found to be 7.1 days, but this was not specific to the *A. aphrophilus* species [5]. While there are a wide range of factors that influence the timing of blood culture results, we believe the example in this case to be a clinically significant deviation from the expected mean. One factor that we speculate was related to the prompt growth of *A. aphrophilus* on day 3 is a high bacterial load either from or leading to the concurrent presence of the abscess. Our literature review yielded a case of *A. aphrophilus* in a patient with superficial temporal abscess and the absence of dental disease, further supporting the notion that *A. aphrophilus* is capable of causing an infection of the head and neck region in an individual such as our patient [9]. Most commonly, abscesses of the head and neck region are caused by oral flora [10]. This is supported by other research that shows that *Streptococcus pyogenes* and *Staphylococcus aureus*, which are prevalent in oral flora, most frequently cause skin and soft tissue abscesses of the head and neck region [11]. 

Among the varying outcomes for HACEK endocarditis, strokes are often indicated with a relative excess of hemorrhagic stroke over embolic stroke [1]. As expected, the presence of a stroke increased the length of stay in our patient as he required ventilatory support among other interventions. HACEK septic emboli were also implicated in higher proportions in individuals with valvular vegetations as seen in our patient. *A. aphrophilus* is typically sensitive to most conventional antimicrobial therapy, as we observed in this case [12]. The treatment of infections with antibiotics can be severely hindered by inadequate source control [13]. Thus, it is vital to conduct a thorough investigation of the skin and oral cavity through a detailed history and physical examination in order to lead to more positive outcomes for patients with IE.

## Conclusions

This case was presented because *A. aphrophilus* is a rare etiological agent for IE patients and uncommonly isolates within three days on blood culture. As seen in this report, *A. aphrophilus* can lead to very poor outcomes and can be difficult to manage. Our literature review yielded no other cases of *A. aphrophilus* infection associated with a superficial abscess in the shoulder. The most up-to-date guidelines stress the pertinence of source control in effectively treating infections and decreasing mortality. This case underlines the importance of paying close attention to localized infections via skin and oral exams when HACEK organisms result relatively quickly on blood cultures. We hope this report elucidates a potential link that will prompt clinicians to do a more thorough workup if they are taking care of a patient with a similar presentation, leading to quicker and more accurate treatment in an individual with HACEK endocarditis.
